# Optimized tissue sample preparation for cryo-electron tomography using serial lift-out

**DOI:** 10.52601/bpr.2025.250035

**Published:** 2026-08-31

**Authors:** Wenjing Du, Junhan Yang, Qiang Guo

**Affiliations:** 1 State Key Laboratory of Membrane Biology, Peking-Tsinghua Center for Life Sciences, Academy for Advanced Interdisciplinary Studies, School of Life Sciences, Peking University, Beijing 100871, China; 2 Changping Laboratory, Beijing 102206, China

**Keywords:** Cryo-ET, Tomography, Cryo-FIB, Tissue, Lift-out, *In situ* structural biology

## Abstract

Cryo-electron tomography (cryo-ET) can provide invaluable insights into subcellular structures in their native environment at molecular resolution. Cryo-focused ion beam (cryo-FIB) has been widely adopted to obtain thin lamellae of cells suitable for tomographic data acquisition. However, its application to tissue samples faces significant limitations. The larger sample size extends milling time, reducing sample preparation throughput. Furthermore, the need for repeated, intricate lift-out procedures adds considerable time and complexity to the workflow. The serial lift-out technique, which can prepare multiple lamellae, increases throughput and better preserves the structural integrity of the tissue sample. Here we outline the workflow of the optimized serial lift-out method and provide a detailed protocol for its implementation, exemplified by a vitrified mouse liver tissue sample.

## INTRODUCTION

Compared to single particle analysis (SPA), cryo-electron tomography (cryo-ET) provides structural insights into macromolecular complexes within their native cellular context (Baumeister 2022; Beck and Baumeister 2016). However, the inelastic scattering of the electron beam critically limits resolution. As the mean free path of the electrons is typically between 300 and 400 nm, most of the cells and tissues are too thick for cryo-ET (Rice *et al*. 2018). Sample thinning is required to achieve an optimal lamella thickness of 150–200 nm (Schiøtz *et al*. 2024b). For cellular samples, thinning is typically performed using cryo-FIB milling, a well-established technique (Rigort *et al*. 2012). Due to their thickness and heterogeneity, reproducibly thinning multicellular tissue samples remains challenging (Chen and Guo 2025).

Cryo-electron microscopy of vitreous sections accommodates thick samples but often introduces compression artifacts along the cutting direction of the knife (Dubochet and Sartori Blanc 2001). To adapt the less damaging cryo-FIB methods for tissue sample, large amounts of the samples need to either be trimmed away, or part of the sample must firstly be extracted in a process referred to as lift-out (Schreiber *et al*. 2018; Zhang *et al*. 2021). At the beginning, one lift-out process, which is time consuming, only yields a single lamella from the area of interest (Schaffer *et al*. 2019; Wu *et al*. 2023). Recent advancements, such as Serial Lift-Out and Serialized On-grid Lift-In Sectioning for Tomography, enable multiple lamellae from a single lift-out, improving throughput and better preserving contextual information (Nguyen *et al*. 2024; Schiøtz *et al*. 2024a). These developments enable cryo-ET to extend its applicability beyond isolated cells, offering unprecedented opportunities to investigate subcellular structures in the context of whole tissues. However, challenges remain, including the need for repeated sample loading/unloading and the fragility of sample attachment to the receiver grid.

In this study, we present an optimized and practical protocol for the preparation of lamellae from tissue samples. A small tissue block is initially lifted-out, subsequently sectioned into multiple lamellae, and thinned individually, thereby significantly reduces processing time for averaged lamellae preparation compared to the conventional lift-out strategy. By improving the reproducibility and efficiency of tissue sample thinning, this protocol facilitates high-quality cryo-ET data acquisition and enables reliable tomogram reconstruction for *in situ* structural analysis of tissue samples.

### Advantages of the protocol

1　This method enhances efficiency and throughput by preparing multiple lamellae from a single lift-out process, reducing milling time.

2　This method consistently produces high-quality lamellae approximately 150 nm thick. Furthermore, the double-sided attachment to the grid bar minimizes the risk of lamellae detachment or cracking during sample transfer, ensuring high-quality tomographic data acquisition.

3　This method enables the preparation of multiple lamellae from the tissue block, preserving more contextual information for subsequent analysis.

### Limitations of the protocol

1　Currently, the lift-out step requires manual intervention, hindering the development of a fully automated, high throughput pipeline of this method.

2　The time required for milling can vary significantly depending on tissue type. For example, samples with high lipid content typically require longer processing times. The current method is optimized for liver tissue and may require optimization for different tissue types.

## OVERVIEW OF THIS PROTOCOL

The method involves several steps. First, a copper block is milled from the half-grid bar at room temperature and attached to the Easylift needle. Next, trench milling is performed around the tissue sample’s region of interest at liquid nitrogen (LN2) temperature. The region of interest is then attached to the copper block and lifted out. Following this, the lifted-out tissue block is sectioned into several lamellae and attached to the receiver grid by double-sided redeposition. Finally, each lamella is thinned to approximately 150 nm. These lamellae are then used for subsequent cryo-ET data acquisition.

## MATERIALS

### Equipment

cryo-FIB/SEM microscope with rotatable stage and needle-based lift-out system (Thermo Fisher Scientific Aquilos2 with Easylift system)

### Materials

O-rings, C-Clips, autogrid tweezers, clipping base/station, clipping tools, cryo grid boxes, grid box opening tool (Thermo Fisher Scientific), large and intermediate size tweezers, duckbill tweezers, mark pens.

### Grids

half-grid (copper, Beijing XXBR., Cat. No. T1109M-3), receiver grid (copper, 100/400 mesh, Beijing XXBR., Cat. No. G100/400).

## PROCEDURE

### Preparations

1　In this procedure, tissue samples are prepared using a well-established method described before (Wu *et al*. 2023). Briefly, a small tissue sample is placed on an EM grid, high-pressure frozen, and then transferred to a liquid ethane-propane mixture (ethane:propane = 36.9%:63.1%) at −170°C, allowing 2-methylpentane to dissolve. The sample grids are stocked in LN_2_ until loading into the cryo-FIB chamber.

2　Ensure the FIB instrument chamber vacuum level is below 4 × 10^−5^ Pa at room temperature. Additionally, verify that nitrogen and argon gas cylinders contain sufficient amounts for the procedure.

3　Activate both electron and ion beams of the FIB system.

4　Open the nitrogen gas cylinder valve and adjust the pressure to 0.2 MPa. In the flow view window, set the flow rate to 190 mg/s. Purge the system for a duration of 20 min to ensure proper gas flow and system readiness.

### Preparation of the copper block (Fig. 1)

**Figure 1:**
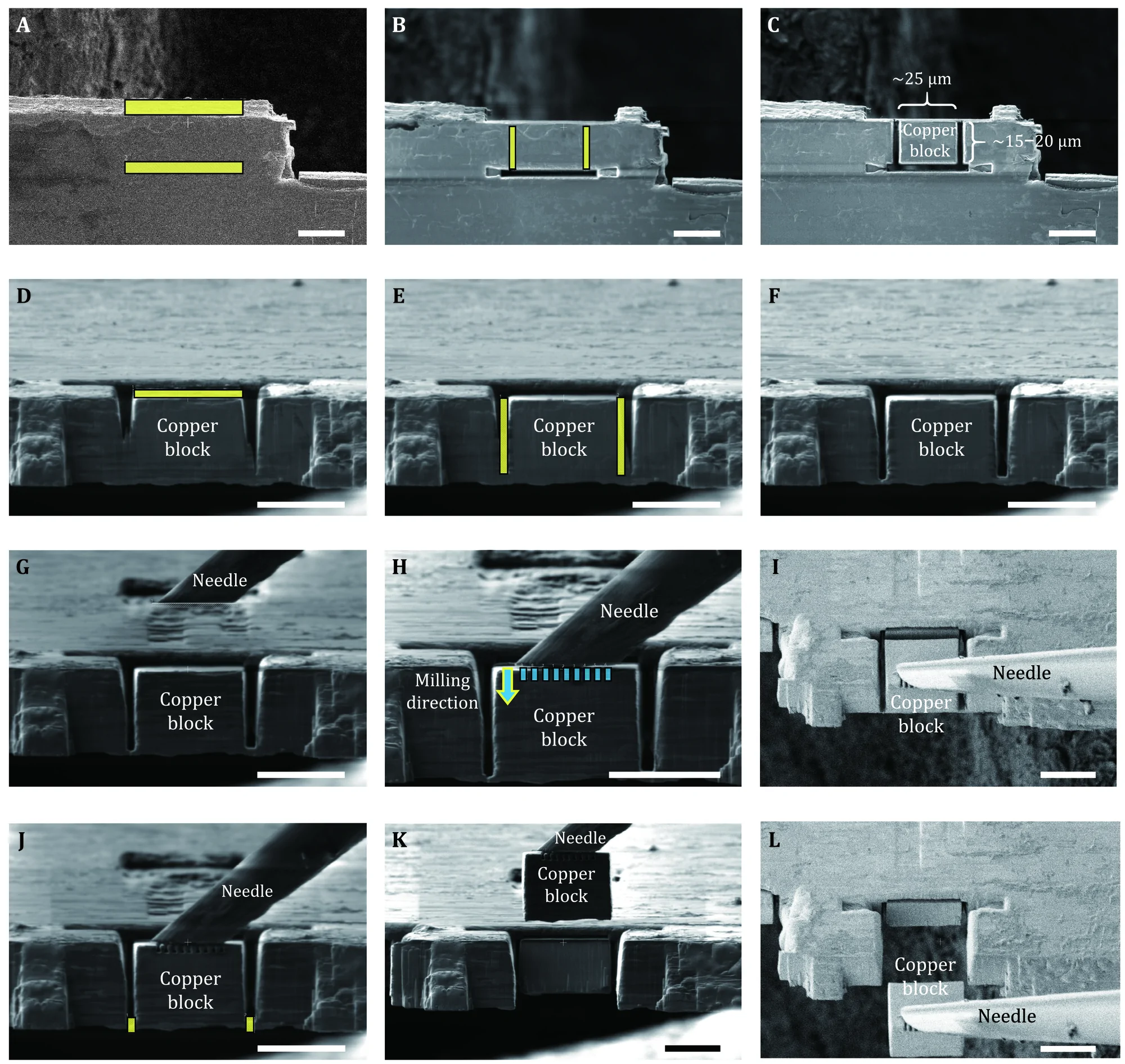
Preparation of the copper block. **A** Trim the edge of the half-grid and mill the region 15 μm away from and parallel to the trimmed edge. **B** Perform perpendicular cuts through the edge at two regions, leaving a little connection on both sides. **C** View from the ion beam after completing steps A and B, showing the defined copper block. **D**,**E** Polish the top surface of the block (**D**) and both sides (**E**). **F** View from the ion beam after polishing the top and sides, showing the flat surfaces. **G** Insert the Easylift needle and polish the needle tip. **H** Attach the needle to the upper surface of the copper block through redeposition milling on the copper block. **I** View from the electron beam after completing redeposition milling. **J** Cut through the remaining connections on both sides of the copper block to release it. **K**,**L** Move the needle away from the half-grid and retract it after the copper block has been successfully released. Rectangle patterns are colored in yellow, regular-cross section patterns are colored in blue. Scale bars, 20 μm

This step should be performed at room temperature, unless a copper block is already attached to the Easylift needle.

1　Perform clipping for a copper half-grid by using a clipping station or clipping base at room temperature. Load an O-ring into the clipping station, followed by placing a copper half-grid in the O-ring groove. Then load a clipping tool with a C-clip and clip the grid into place.

2　Load the clipped half-grid in Grid Shuttle with the edge parallel and the filled half facing downward. Then load the shuttle into the cryo-FIB stage.

3　Change the stage position to the trench milling position (Stage rotation: 110°, Stage tilt: 7°). Focus on the edge of the half-grid and link the stage Z to FWD. Use a rectangular pattern with a beam current of 30 nA (30 kV, same for subsequent steps) and a Z-depth of 10 μm to trim the edge. Mill the region 15 μm away from and parallel to the trimmed edge (Fig. 1A).

4　Perform perpendicular cuts at two regions using the same pattern above, leaving a little connection on both sides (Fig. 1B). This creates a copper block with dimensions of 20 µm in length and 15 µm in width (Fig. 1C). If necessary, polish the block surfaces with a rectangle pattern with a 5 nA beam current.

5　Next, adjust the stage position to the lamella milling position (Stage rotation: −70°, Stage tilt: 18°). Use a rectangle pattern with a beam current of 5 nA to polish the top and both sides of the block (Figs. 1D, 1E and 1F).

6　Ensure the stage is in a safe position before inserting the Easylift needle and positioning it near the top of the copper block. Remove residual copper block attached to the needle and polish the needle tip using a rectangle pattern with a beam current of 5 nA. Leave a minimum of 10 µm of the needle surface exposed at the base and ensure it remains flat (Fig. 1G).

7　Attach the needle to the upper surface of the copper block through redeposition milling on the copper block directed away from the attachment site. Position the milling start of the patterns at the interface between the needle and the copper block (Figs. 1H and 1I). Use a regular cross-section pattern (width: 1 μm, height: 2 μm, lateral spacing: 1 μm, beam current: 0.5 nA, Z-depth: 5 μm, multi-passes: 1).

8　Cut through the remaining connections on both sides of the copper block using a rectangle pattern with a beam current of 5 nA (Fig. 1J). Verify detachment of the copper block by slightly moving the needle. Once detached, move it away from the half-grid and retract the needle (Figs. 1K and 1L).

### Loading of the sample grid and the receiver grid

1　Cool down the instrument and the loading station with dry LN_2_.

2　For the receiver grid, perform clipping at room temperature using the same procedure as described for the copper half-grid. Then mark the Autogrid at three positions. One marking is placed in line with the 400-mesh grid bars and two other markings are placed in line with the 100-mesh grid bars.

3　For the sample grid, perform clipping at LN_2_ temperature, ensuring the tissue side faces downward. Prior to clipping, mark the O-ring at the 9, 3, and 6 o’clock positions with black markers.

4　Load the sample grid in position 1 of the Grid Shuttle and the receiver grid in position 2, ensuring the three markings (3, 6, and 9 o’clock) are aligned in their correct orientations. Then load the shuttle into the cryo-FIB stage.

### Trench milling (Figs. 2A, 2B, 3A, 3B)

**Figure 2:**
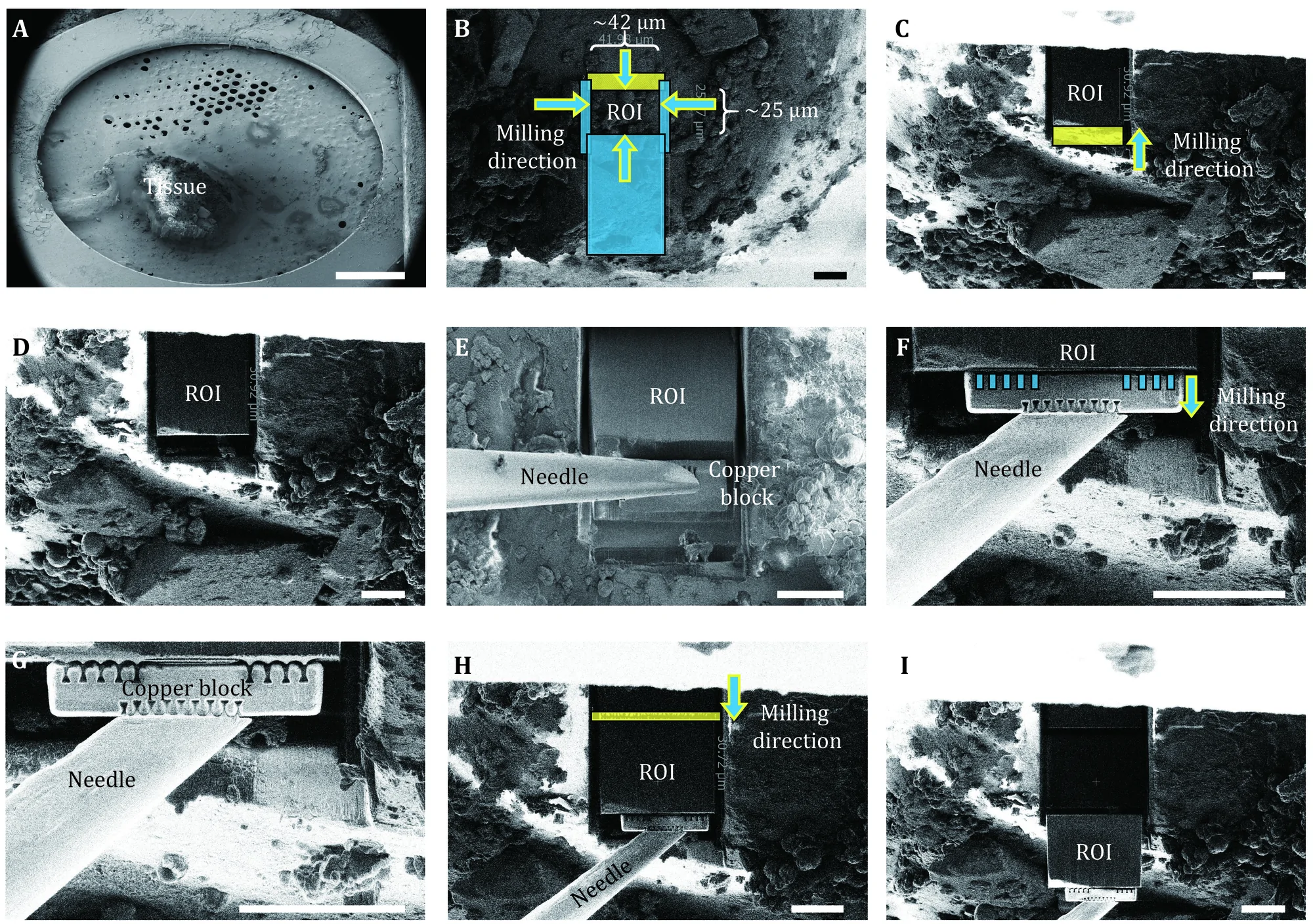
Trench milling and lift-out. **A** The overview image of the sample grid after depositing a protective Pt layer and subsequent sputter coating. **B** Milling of four trenches around the region of interest (ROI). **C** Polishing of the top face of the tissue block. **D** View from the ion beam after polishing the top face of the tissue block. **E** Maneuver the needle to make it contact with the tissue block. **F**,**G** Attaching the copper block to the tissue block by redeposition milling on the copper block. **H** Cutting through the lower part of the tissue block to release it from the remaining tissue. **I** After detachment, move the needle along with the tissue block to an open area and retract it. The tissue block is successfully lifted out. Rectangle patterns are colored in yellow, regular-cross section patterns are colored in blue. Scale bars, 500 μm for panel A and 20 μm for all other panels

**Figure 3:**
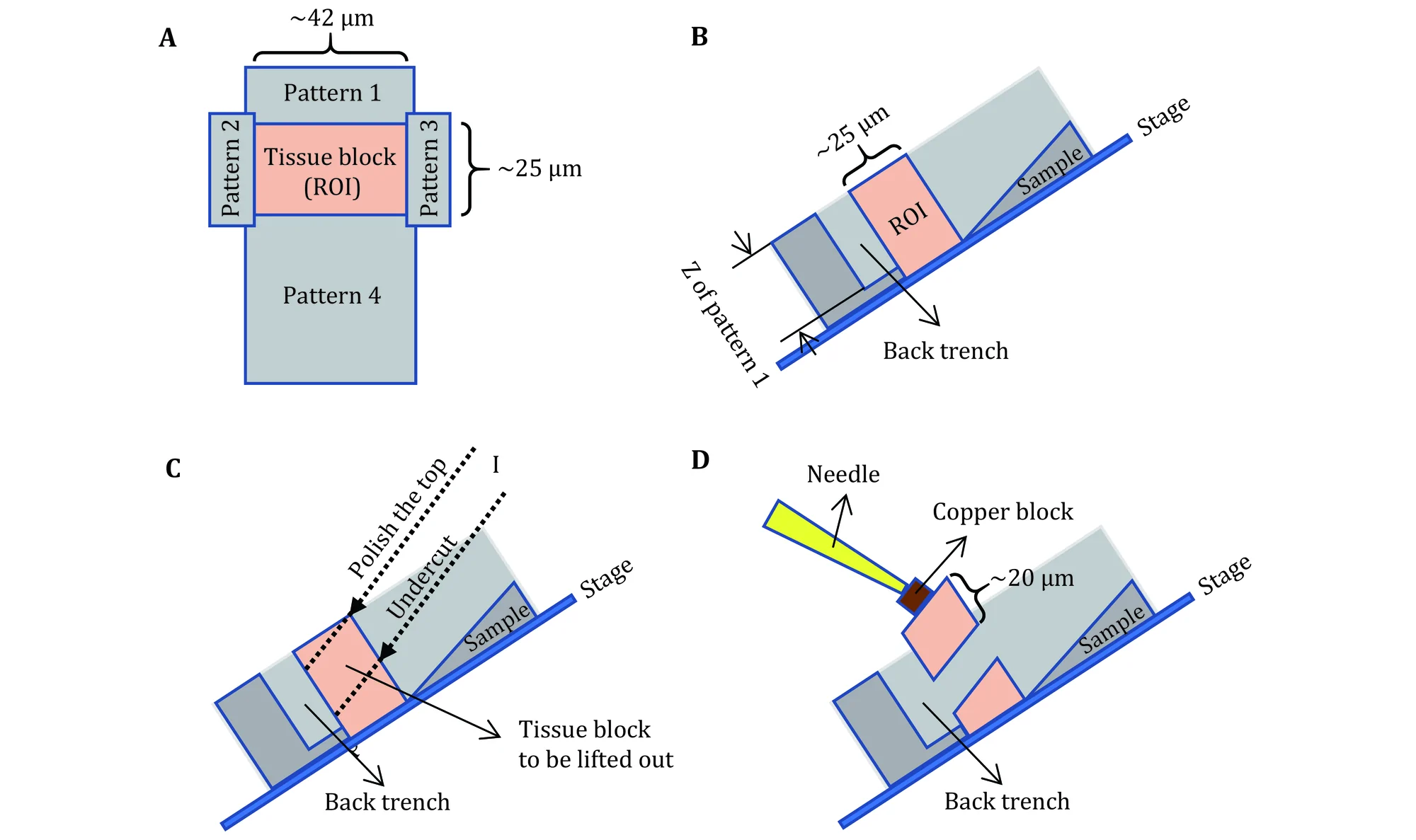
A schematic of trench milling and lift-out procedure. **A** The positions of four patterns around the region of interest (ROI). **B** The side view of the ROI after trench milling. **C** The positions for top surface polishing and undercutting. **D** The step of lift-out after redeposition and undercut

1　Navigate to the sample grid. Focus on the sample and link the stage Z to FWD.

2　Change the stage position to the deposition position (Stage rotation: −70°, Stage tilt: 45°). Deposit a protective Pt layer by inserting and opening the GIS (Gas Injection System) needle for 30 s (depend on specific machines, the optimal thickness of protective Pt layer is about 1.0 μm). Then the GIS needle is retracted and a conductive Pt layer is deposited with the Sputter Coating System (time: 15 s, current: 30 mA) (Fig. 2A).

3　Change the stage position to the trench milling position (Stage rotation: 110°, Stage tilt: 7°). Set the scan rotations of both electron and ion beam to 0°. Navigate to the region of interest (ROI) on the tissue block, focus and link the stage. If an integrated fluorescence light microscope (iFLM) is available, it can further assist in localizing target proteins via fluorescence imaging. Use a combination of milling patterns to mill the trenches (Figs. 2B, 3A and 3B):

(A) Back trench: apply a rectangular pattern with a beam current of 3 nA and a Z-depth of 4 μm.

(B) Side and front trenches: apply regular cross-section patterns with a beam current of 3 nA and a Z-depth of 4 μm.

4　Mill four trenches surrounding the ROI, leaving a tissue block approximately 25 μm in length and 42 μm in width. Ensure the width is at least 2 μm greater than the spacing between two adjacent 400-mesh grid bars of the receiver grid.

### Lift-out (Figs. 2C–2I, 3C, 3D)

1　Change the stage position to the lamella milling position (Stage rotation: −70°, Stage tilt: 18°). Examine the front face of the tissue block for accessibility. If it is not accessible, return to the trench milling position to mill the front trench further. If accessible, polish the top face of the tissue block using a rectangle pattern with a beam current of 1 nA (Figs. 2C, 2D and 3C).

2　Insert the Easylift needle and position it near the tissue block. Polish the bottom face of the copper block if necessary. Then move the needle to bring the bottom face of the copper block into contact with the top face of the tissue block (Fig. 2E).

3　Attach the copper block to the tissue block by redeposition milling on the copper block directed away from the attachment site. Position the start of the milling patterns on the interface between the tissue block and the copper block (Figs. 2F and 2G). Use a regular cross-section pattern (width: 1 μm, height: 2 μm, lateral spacing: 1 μm, beam current: 0.5 nA, Z-depth: 5 μm, multi-passes: 1).

4　Cut through the lower part of the tissue block using a line pattern (Z-depth: 20 μm, beam current: 1 nA) to release it from the surrounding tissue. For challenging tissues, use a rectangle pattern (Z-depth: 20 μm, beam current: 3 nA) to confirm complete detachment (Figs. 2H and 3C). Slightly move the needle laterally to verify the release. If the tissue block remains attached to the surrounding tissue, continue milling until detachment is achieved.

5　Once detached, move the needle carrying the tissue block to an open area and retract the needle (Figs. 2I and 3D).

### Sample transfer and sectioning (Figs. 4A–4F)

**Figure 4:**
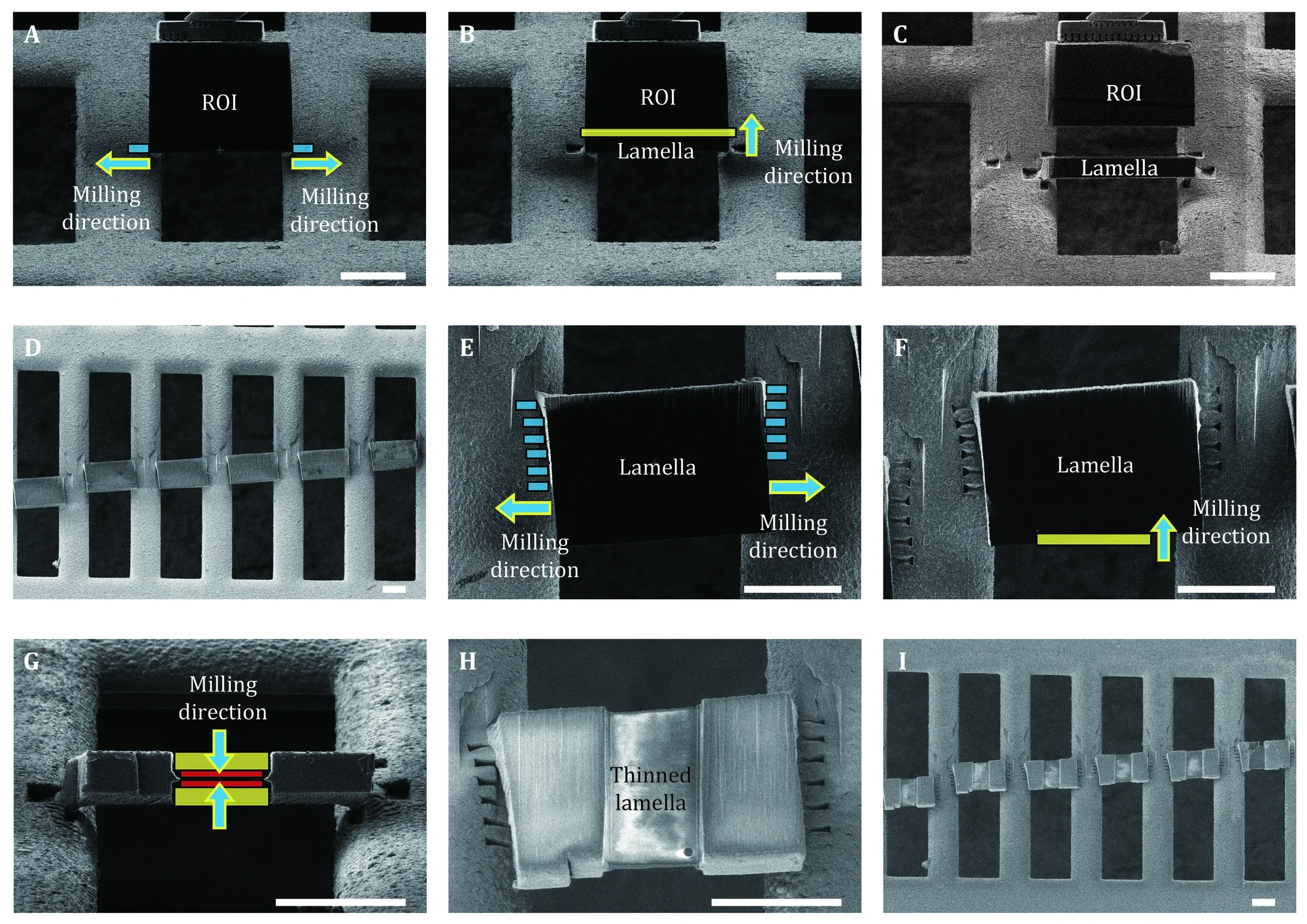
Sample transfer, sectioning and thinning. **A** Attach the tissue block to the grid bars by redeposition milling on the grid bars. **B** Section the tissue block to create a lamella. **C** Move the needle away and retract it after successfully releasing the tissue block. **D** View from the electron beam after completely sectioning. **E** Perform additional redeposition milling on both grid bars to solidate the initial attachment and ensure stability. **F** Polish the front surface of each lamella. **G** Thin the lamella using the rectangle and cleaning cross-section patterns. **H** View from the electron beam after thinning. **I** View from the electron beam showing that all lamellae have been successfully thinned. Rectangle patterns are colored in yellow, regular-cross section patterns are colored in blue, cleaning cross-section patterns are colored in red. Scale bar, 20 μm

1　Navigate to the receiver grid. Change the stage position to the lamella milling position (Stage rotation: −70°, Stage tilt: 18°). Set the scan rotations of both the electron and the ion beam to 180°. Finely adjust the stage position to ensure the 400-mesh grid bars are aligned vertically in the image.

2　Navigate to the space between two adjacent 400-mesh grid bars and insert the needle after focusing and linking the stage. Align the edges of the tissue block with the grid bars. If the tissue block exceeds the required dimensions, mill off the excess material. Precisely adjust the needle to bring the tissue block in contact with the two adjacent grid bars (Fig. 4A).

3　Attach the tissue block to the grid bars by redeposition milling along both grid bars with regular cross-section patterns (width: 6 μm, height: 2 μm, beam current: 0.5 nA, Z-depth: 5 μm, multi-passes = 1) directed away from the tissue block (Fig. 4A).

4　Mill the tissue block approximately 3–4 μm from the lower edge using a line pattern (Z-depth: 20 μm, beam current: 1 nA) to create a lamella. For challenging tissues, a rectangle pattern (Z-depth: 20 μm, beam current: 3 nA) may be employed (Fig. 4B). Verify that the remaining tissue block has been released from the lamella by gently moving the needle.

5　Once the tissue block is released, retract the needle (Fig. 4C). Adjust the stage to the next position between two adjacent 400-mesh grid bars. Resume the process from Step 2 of this Part, and repeat until the entire tissue block has been sectioned and sequentially attached to the grid bars (Fig. 4D).

6　Adjust the stage to the redeposition consolidation position (Stage rotation: 180° relative to the adjusted lamella milling position, Stage tilt: 7°). Set the scan rotations of both electron and ion beam to 0°. Perform redeposition milling on both grid bars for each lamella using regular cross-section patterns (width: 4 μm, height: 1.5 μm, beam current: 0.5 nA, Z-depth: 5 μm, multi-passes = 1) directed away from the tissue block (Fig. 4E).

7　At the redeposition consolidation position, polish the front surface of each lamella using a rectangle pattern (Z-depth: 3 μm, beam current: 0.5 nA) to make it properly flat (Fig. 4F).

### Lamella thinning (Figs. 4G–4I)

1　Adjust the stage to the deposition position (Stage rotation: −70°, Stage tilt: 45°). Deposit a protective Pt layer by inserting and opening the GIS needle for 30 s. Then the GIS needle is retracted and a conductive Pt layer is deposited with the Sputter Coating System (time: 7 s, current: 15 mA).

2　Adjust the stage to the lamella milling position established in Step 1 of the previous part. Ensure that the 400-mesh grid bars are vertically aligned in the image. Use a rectangle pattern to thin each lamella for rough milling until reaching a thickness of approximately 300 nm. Decrease the beam current as the lamella thickness reduces (Fig. 4G). This step is similar to milling the cell described by Wegner *et al.* (Wagner *et al*. 2020).

3　Tilt the stage by ±0.5° and use a cleaning cross-section pattern (Z-depth: 10 μm, beam current: 30 pA) to ensure uniform thickness by milling the back of the lamella. Return the stage to the original tilt and continue milling to achieve a final thickness of approximately 150 nm. The final thinned area should have a width of around 10–15 μm. Proceed to the next lamella and repeat the thinning process (Figs. 4H and 4I).

4　If available, employ the AutoTEM Cryo software to automate Step 2 of this part, followed by manual polishing using a beam current of 30 pA for fine adjustments. The uniform size of all lamellae allows for the effective use of automated thinning. For instance, the milling program can be set similarly to the cell samples.

(A) The thinning pattern dimensions should be adjusted to the size of the lamellae, for instance, a width of 10–15 μm and a height appropriate for the current lamella thickness. The time required for each thinning step depends on sample properties and instrument conditions, and should be fine-tuned empirically.

5　After thinning all lamellae, perform additional sputter coating (current: 7 mA, time: 7 s) to reduce sample charging during data acquisition and help image alignment.

### Completion

Acquire a low-magnification SEM image of all thinned lamellae to confirm their integrity (Fig. 4I). Sleep both electron and ion beams. Carefully transfer the sample grids and receiving grids back into the grid box and store them in LN_2_. Remove the heat exchanger to allow the microscope to return to room temperature. Refill the LN_2_ tank, and close the nitrogen gas valve.

## ANTICIPATED RESULTS

**Figure 5:**
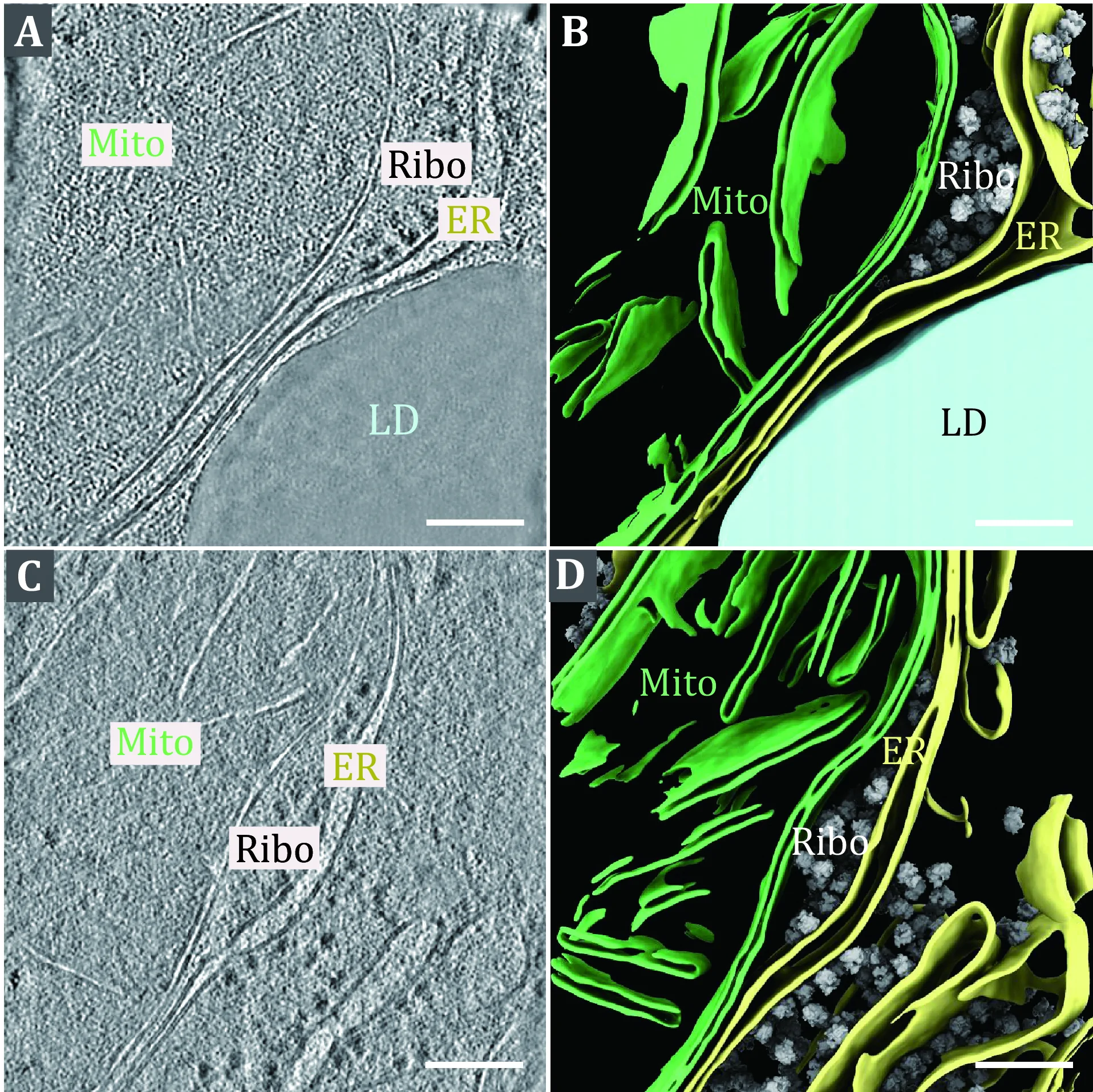
Representative tomographic data collected from the thinned lamellae. **A**,**C** 1.1 nm thick tomographic slice of a representative cryo-ET tomogram collected from the hepatocyte region of mouse liver sample. **B**,**D** 3D renderings of the segmented structures from the tomograms shown in Panels A and C, respectively. mito: mitochondrion. ribo: ribosome. ER: endoplasmic reticulum. Mitochondria, ER, ribosomes and lipid droplet are colored in green, yellow, grey and magenta, respectively. Scale bars, 100 nm

This protocol should yield around seven lamellae suitable for high-resolution tilt series acquisition in a day. Alternatively, the process can be conducted over two days: day one for trench milling, lift-out and sectioning, and day two for thinning. The sample can be stored in LN2 after lift-out and sectioning, prior to thinning. This approach should yield 16 or more lamellae over two days. These lamellae enable the reconstruction of tomograms after cryo-ET data acquisition, providing detailed visualization of the tissue’s subcellular architecture, as demonstrated in our recent study of mouse liver (Fig.5) (Wu *et al*. 2024). Averaging the extracted subtomograms can subsequently yield *in situ* reconstructions of protein complexes, such as ribosomes, proteasomes, and microtubules. These reconstructions can achieve nanometer-scale resolutions or better, while preserving spatial distribution information and accurately reflecting the native states of these protein complexes within tissues.
